# Transurethral resection syndrome in elderly patients: a retrospective observational study

**DOI:** 10.1186/1471-2253-14-30

**Published:** 2014-04-23

**Authors:** Junko Nakahira, Toshiyuki Sawai, Atsushi Fujiwara, Toshiaki Minami

**Affiliations:** 1 Department of Anesthesiology, Osaka Medical College, 2-7 Daigaku-machi, Takatsuki, Osaka, Japan

**Keywords:** TUR syndrome, Hyponatremia, Transurethral resection of prostate, Irrigation fluid

## Abstract

**Background:**

Transurethral resection of the prostate (TURP) involves the risk of transurethral resection (TUR) syndrome owing to hyponatremia. Irrigation fluid type, duration of operation, and weight of resected mass have been evaluated as risk factors for TUR syndrome. The purpose of the present study was to identify risk factors related to TUR syndrome in the elderly.

**Methods:**

After obtaining approval from the Institutional Review Board, data on all elderly males (aged 70 years and older) who underwent TURP under regional anesthesia over a 6-year period at our institution were retrospectively reviewed.

TUR syndrome was defined as evidence of a central nervous system disturbance such as nausea, vomiting, restlessness, confusion, or even coma with a circulatory abnormality both intra- and post-operatively. Patients were divided into two groups, positive and negative, for the occurrence of the syndrome. Data such as previous medical history, preoperative and postoperative serum data, weight of resected mass, duration of operation, irrigation fluid drainage technique, anesthetic technique, operative infusion and transfusion volume, and neurological symptoms were collected. Only observational variables with p < 0.05 on univariate analyses were included in the multivariate logistic regression model to ascertain their independent effects on TUR syndrome.

**Results:**

Of the 98 patients studied, 23 had TUR syndrome (23.5%, 95% confidence interval [CI] 14.9–32.0%). Multivariate regression analysis revealed that volume of plasma substitute ≥ 500 ml (odds ratio [OR] 14.7, 95% CI 2.9–74.5), continuous irrigation through a suprapubic cystostomy (OR 4.7, 95% CI 1.3–16.7), and weight of resected mass > 45 g (OR 4.1, 95% CI 1.2–14.7) were associated with significantly increased risks for TUR syndrome (Hosmer-Lemeshow test, p = 0.94, accuracy 84.7%).

**Conclusions:**

These results suggest that the use of a plasma substitute and continuous irrigation through a suprapubic cystostomy must be avoided during TURP procedures in the elderly.

## Background

Benign prostatic hyperplasia is common in elderly males. Transurethral resection of the prostate (TURP) is a standard surgical procedure for the management of benign prostatic obstructions. Non-conductive irrigation fluid is required during the use of monopolar electric resectoscope to clear the operating field. This hypotonic, electrolyte-free, nonconductive distension solution contains no electrolytes, and excessive absorption of it can cause fluid overload and dilutional hyponatremia. The associated adverse effects arising in both the cardiovascular and nervous systems are known as transurethral resection (TUR) syndrome. TUR syndrome has a multifactorial pathophysiology that is now better understood but still remains a risk.

Several studies over the last 20 years have shown mortality rates of 0.2–0.8% [1], and TURP is still associated with significant morbidity [2,3]. The most frequent complication of conventional TURP is perioperative bleeding, which, in a significant number of cases, may necessitate blood transfusion. The most serious complication of conventional monopolar TURP is TUR syndrome, the frequency of which varies considerably in the literature, ranging from 0.18 to 10.9% [4,5]. The symptoms of TUR syndrome are central nervous disturbances such as dizziness, headache, nausea, vomiting, and apnea, and circulatory abnormalities such as hypertension, hypotension, bradycardia, and arrhythmia. Anesthesiologists need to remain vigilant for such signs of TUR syndrome during surgery. Left undiagnosed, this syndrome can lead to lung or cerebral edema [6]. Therefore, spinal anesthesia is often recommended for TURP procedures so that early signs of neurological deterioration can be detected.

TUR syndrome can occur during other operations, including transcervical resection of the endometrium, TUR of bladder tumors, cystoscopy, arthroscopy, and vesical ultrasonic lithotripsy. However, TURP has an extremely high incidence of TUR syndrome. Theoretical risk factors are opened prostatic sinuses, high irrigation pressures, lengthy resection, and hypotonic irrigation solutions [7]. According to a past report, 77% of patients undergoing TURP had significant pre-existing medical conditions. Increased morbidity was found in patients with a resection time greater than 90 min, gland masses greater than 45 g, acute urinary retention, age greater than 80 years, and in those of African descent [8]. The aim of the present study was to identify risk factors related to TUR syndrome in the elderly.

## Patients and methods

After obtaining approval from the Ethical Committee of Osaka Medical College, data on all elderly males (aged 70 years and older) who underwent TURP with regional anesthesia from April 2006 to March 2011 at our institution were retrospectively reviewed. Spinal anesthesia (L3/4 or L4/5) and epidural tubing (L1/2 or L2/3) were administered before the operations. 0.5% hyperbaric bupivacaine hydrochloride hydrate (1.8–3.2 ml) as a spinal anesthetic was used to obtain analgesia up to the T (Thoracic) 10 level. Cases of failed spinal anesthesia converted to general anesthesia were excluded from the analysis. If the T levels were lower or the operation time continued over 1.5 h, 0.375% ropivacaine hydrochloride (3.0–5.0 ml) was administered via the epidural tube. Postoperative analgesia was obtained using continuous epidural anesthesia of 2–5 ml/h of 0.2% ropivacaine. The surgical interventions were performed with monopolar electronic retroscope by surgeons with the same qualifications and clinical experience. D-sorbitol 3% was used as the nonconductive irrigation fluid. Bags were placed 90 cm above the operating table. Hemodynamic monitoring included heart rate, electrocardiogram, systolic and diastolic blood pressures every 2 min, and percutaneous oxygen saturation. Exclusion criteria included patients with bleeding disorders or existing coagulopathy and renal insufficiency, as well as any contraindication to spinal anesthesia. All patients were preloaded with an infusion of lactated Ringer’s fluid before induction of spinal anesthesia.

TUR syndrome was defined as the presence of central nervous system disturbances such as nausea, vomiting, restlessness, pain, confusion, or even coma with circulatory abnormalities both intra- and post-operatively. A checklist recommended by Hahn et al. to grade symptoms was used (Table 1) [9]. The presence of at least one circulatory disorder and one neurological disorder is necessary for a diagnosis of TUR syndrome. For circulatory abnormalities such as hypertension (>30% above baseline systolic blood pressure), hypotension (systolic blood pressure <80 mmHg), bradycardia, and arrhythmia, immediate treatment was prepared to avoid deterioration. For systolic blood pressures less than 80 mmHg, 4 mg of ephedrine hydrochloride was immediately administered intravenously. Medical and nursing personnel were intimately involved in patient care, in the monitoring and assessment of complications and the incidence and severity of TUR syndrome in the postoperative period.

**Table 1:** Severity score

	**Severity score**		
	**1**	**2**	**3**
Circulatory			
Chest pain	Duration < 5 min	Duration > 5 min	Repeated attacks
Bradycardia	HR decrease 10–20 bpm	HR decrease > 20 bpm	Repeated decreases
Hypertension	SAP up 10–20 mmHg	SAP up > 30 mmHg	Score (2) for 15 min
Hypotension	SAP down 30–50 mmHg	SAP down > 50 mmHg	Repeated drops > 50 mmHg
Poor urine output	Diuretics needed	Repeated use	Diuretics ineffective
Neurological			
Blurred vision	Duration < 10 min	Duration > 10 min	Transient blindness
Nausea	Duration < 5 min	Duration 5–120 min	Intense or > 120 min
Vomiting	Single instance	Repeatedly, < 60 min	Repeatedly, > 60 min
Uneasiness	Slight	Moderate	Intense
Confusion	Duration < 5 min	Duration 5–60 min	Duration > 60 min
Tiredness	Patient says so	Objectively exhausted	Exhausted for > 120 min
Consciousness	Mildly depressed	Somnolent < 60 min	Needs ventilator
Headache	Mild	Severe < 60 min	Severe > 60 min

A checklist used to define and score symptoms included in the TUR syndrome [9]. HR, heart rate; SAP, systolic arterial pressure.

**Figure 1:**
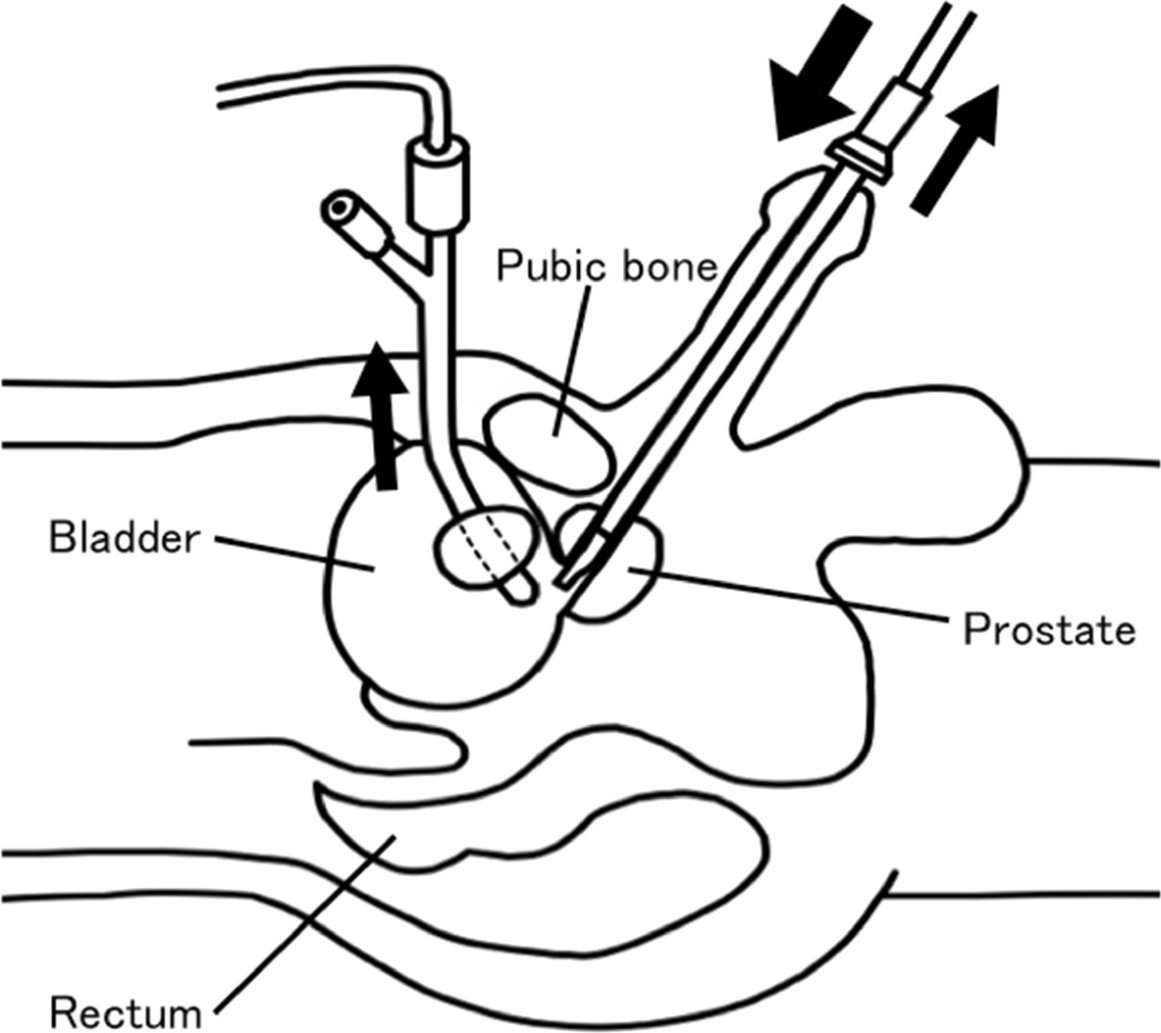
**Continuous irrigation fluid drainage through suprapubic cystostomy.** A catheter of the irrigation fluid drainage was inserted preoperatively under spinal anesthesia. Arrows demonstrate flows of the irrigation fluid.

Patients were divided into two groups, positive and negative, for the occurrence of the syndrome, and their risk factors were evaluated accordingly. Observational parameters were patient characteristics, dosage of local anesthesia, duration of the operation, weight of resected mass, volume of Ringer’s fluid, volume of plasma substitute, and whether continuous drainage of irrigation fluid through a suprapubic cystostomy via a pigtail drainage catheter (Angiomed Gmbh & Co. Medizintechnik Kg, Karlsruhe, Germany) was performed (Figure 1). Hespander (Fresenius Kabi Japan, Tokyo, Japan), which contains hydroxyethyl starch 6.0 g/100 ml, was used for the plasma substitute. Only variables with p < 0.05 on univariate analyses were included in the multivariate logistic regression model to determine their correlation with TUR syndrome. Whether or not the patients became symptomatic, adequate therapeutic measures were taken to prevent further complications. Timing of blood samples was at the discretion of the anesthesiologists or surgeons.

### Statistical analysis

Univariate logistic regression analyses using parameters judged to be risk factors for TUR syndrome in the literature [1], including age, body weight, and operating time, etc., were performed. Only observational variables with p < 0.05 in univariate analyses were included in the multivariate logistic regression model to ascertain their independent effects on TUR syndrome. The odds ratio and p values were calculated for each variable. A p value of < 0.05 was considered significant. Analysis was performed using the statistical analysis software SPSS version 17.0 for Windows (SPSS, Chicago, IL, USA).

## Results

A total of 98 patients with a median age of 76 (range, 70–91) years was included in this study. Of these, 23 had TUR syndrome (23.5%, 95% CI 14.9–32.0%). Initial circulatory abnormalities were mainly hypertension with reflex bradycardia, or sudden hypotension. In terms of patient characteristics and preoperative data, there were no significant differences between the two groups (Table 2). For grading symptoms, the checklist was used. The severity score of all of the patients with TUR syndrome was 2 and greater at the end of the procedure. Patients with a score of 3 were handled using additional anesthetic agents such as propofol or midazolam administered intravenously to support respiratory status and relieve pain immediately. In six of the 23 TUR syndrome patients, additional anesthesia including drugs for nausea was needed owing to the severity of their symptoms. One of the TUR syndrome patients was intubated owing to the severity of cardiovascular and neurological symptoms. One patient without TUR syndrome had an esophageal hemorrhage after the procedure because of esophageal variceal rupture. One patient without TUR syndrome during the procedure had postoperative nausea and vomiting. All transfusions were preoperative autologous blood donations except for one patient with TUR syndrome, who received allogeneic transfusion of red blood cells.

**Table 2:** Patients’ characteristics

	**TUR syndrome**		
**Parameter**	**Yes (n = 23)**	**No (n = 75)**	**p value**
Age, years	77.5 ± 5.2	75.4 ± 4.5	0.115
Height, cm	163.9 ± 6.5	162.8 ± 5.6	0.414
Body weight, kg	61.4 ± 9.3	60.6 ± 7.9	0.296
Diabetes mellitus	3 (13.0%)	12 (16.0%)	0.512
Hypertension	4 (17.4%)	11 (14.7%)	0.488
CRF	1 (4.3%)	2 (2.7%)	0.556
Cardiac disease	0 (0.0%)	2 (2.7%)	0.081
Preoperative serum data			
Creatinine	0.9 ± 0.4	0.9 ± 0.4	0.612
BUN, g/dl	15.9 ± 6.8	16.7 ± 8.2	0.802
Sodium, mol	140.6 ± 2.4	140.3 ± 2.8	0.446
Hemoglobin, g/dl	13.5 ± 1.1	13.6 ± 1.7	0.837
Hematocrit,%	39.1 ± 3.2	39.6 ± 4.9	0.819

Data expressed as means ± SD or number (%). TUR syndrome was defined as the presence of central nervous system disturbances such as nausea, vomiting, restlessness, pain, confusion, or even coma with circulatory abnormalities both intra- and post-operatively.

CRF, chronic renal failure; BUN, blood urea nitrogen.

Duration of operation ≥ 1.5 h, weight of resected mass > 45 g, volume of plasma substitute ≥ 500 ml, and continuous irrigation through a suprapubic cystostomy were significantly associated with TUR syndrome on univariate analyses (Table 3). Multivariate regression analysis showed that weight of resected mass > 45 g, volume of plasma substitute ≥ 500 ml, and continuous irrigation were associated with a significantly increased risk for TUR syndrome (Table 4).

**Table 3:** Operative and postoperative data

	**TUR syndrome**		
**Parameter**	**Yes (n = 23)**	**No (n = 75)**	**p value**
Continuous irrigation fluid drainage	14 (60.9%)	12 (16.0%)	< 0.001
0.5% Bupivacaine, ml	2.4 ± 0.4	2.4 ± 0.4	0.979
Resection weight, g	56.2 ± 31.0	30.8 ± 24.9	0.251
Resection weight > 45 g	14 (60.9%)	13 (17.3%)	< 0.001
Operation time, min	106 ± 39	70 ± 27	0.041
Operation time ≥ 1.5 hours	15 (65.2%)	19 (25.3%)	< 0.001
Plasma substitute, ml	367 ± 523	21 ± 99	< 0.001
Plasma substitute	11 (47.8%)	4 (5.4%)	< 0.001
Plasma substitute ≥ 500 ml	9 (39.1%)	3 (4.0%)	< 0.001
Saline, ml	200 ± 36	123 ± 149	0.005
Ringer fluid, ml	426 ± 329	431 ± 241	0.396
Symptom	23 (100.0%)	NA	NA
Restlessness	14 (60.9%)	NA	NA
Vomiting	11 (47.8%)	NA	NA
Nausea	8 (34.8%)	NA	NA
Pain	15 (65.2%)	NA	NA
Transfusion	16 (69.6%)	15 (20.3%)	< 0.001
Diuretics	4 (17.4%)	3 (4.0%)	0.051
Sodium chloride, mol	1.4 ± 3.3	0.2 ± 1.3	< 0.001
Postoperative serum data			
Creatinine, mg/dl	1.0 ± 0.6	1.0 ± 0.4	0.382
BUN, mg/dl	13.5 ± 7.4	14.3 ± 9.0	0.839
Sodium, mEq/l	132.7 ± 8.4	137.6 ± 3.8	< 0.001
Hemoglobin, g/dl	10.7 ± 1.4	12.5 ± 1.8	0.081
Hematocrit, %	31.4 ± 4.0	36.1 ± 5.4	< 0.001

Data expressed as mean ± SD or the number of patients (%). TUR syndrome was defined as the presence of central nervous system disturbances such as nausea, vomiting, restlessness, pain, confusion, or even coma with circulatory abnormalities both intra- and post-operatively. NA, not applicable; TUR, transurethral resection; BUN, blood urea nitrogen.

**Table 4:** Multivariate analysis

**Parameter**	**Odds ratio**	**95% Confidence interval**	**p value**
Plasma substitute ≥ 500 ml	14.7	2.9–74.5	0.001
Continuous irrigation fluid drainage	4.7	1.3–16.7	0.018
Resection weight > 45 g	4.7	1.2–14.7	0.029

Accuracy 84.7%, Hosmer-Lemeshow test, p = 0.944.

## Discussion

In the present study, TUR syndrome was defined as the presence of one or more cardiovascular symptoms and one or more neurological symptoms. In previous reports, the occurrence of TUR syndrome varied, with a range of 0.5 to 10.5%, because few studies used a clear definition. Many previous studies defined TUR as sodium concentrations of 125 mmol/l or less after TURP with two of the following symptoms: nausea, vomiting bradycardia, hypotension, chest pain, mental confusion, anxiety, paresthesiae, and visual disturbance [10]. According to our clinical data and experience, not all patients with dynamic cardiovascular and neurological symptoms have serum sodium levels of 125 mmol/l or less. When a patient has only one symptom, the serum sodium level can be more than 135 mmol/l. In the present study, the rate of incidence of TUR syndrome was higher than in past reports. There are two possible reasons for this. One is the advanced age of the patients in the present study, because circulatory abnormalities are a greater risk in elderly patients. Another possible reason is that serum sodium levels were not included in the present definition of TUR syndrome. As mentioned, the serum sodium level is even more than 135 mmol/l at the beginning of TUR syndrome. If the patient’s status deteriorated, treatment was performed to avoid the occurrence of more severe symptoms. With respect to other conditions, D-sorbitol 3% was used as the nonconductive irrigation fluid. Bags were placed 90 cm above the operating table. Hahn et al. reported no significant differences in the volume of irrigation fluid absorbed at different bag heights [1]. The safe height for the irrigation fluid during TURP remains controversial, but it may affect the quantity of fluid absorbed.

As many previous studies showed, significant risk factors for TUR syndrome were duration of operation > 1.5 h and weight of resected mass > 45 g [8]. Despite all technical advances, resection speeds of between 0.5 and 0.9 g/min have not shown significant improvement [6]. In contrast, Akata et al. reported that changes in serum sodium during TURP correlated with rapid absorption in cases when the capsular veins and the prostate sinus are injured, but not with resection times [11]. Volume of plasma substitute ≥ 500 ml during the operation was found to be a significant risk factor for TUR syndrome in the present study. Plasma substitute contains colloids with a slightly higher osmotic pressure than Ringer’s solution or saline and it is administered from the beginning of the surgery against hypotension caused by spinal anesthesia [12]. Theoretically, excessive serum dilution should have been avoided.

More importantly, continuous drainage of irrigation fluid through a suprapubic cystostomy was found to be another risk factor in the present study. Continuous fluid drainage is useful for removing debris and blood in the operating field and in removing postoperative blood clots. This allows surgeons to continue the procedure without interruption. However, according to our observations, a number of patients had abdominal swelling owing to irrigation fluid leaking from the drainage point into the extraperitoneal space and abdominal cavity. Interestingly, Hahn et al. reported on evacuation of irrigation fluid from the bladder. Keeping the intravesical pressure below 2 kPa is the best strategy for reducing fluid absorption. The drainage catheter may not have been effective because it had a small diameter [13]. Extracellular electrolytes diffuse into the deposited irrigating fluid [14]. Abdominal pain, which may radiate to the shoulder, is a common first sign of extravasation [15]. Then, even if a suprapubic cystostomy should be inserted into the extraperitoneal area, the peritoneum can absorb large amounts of leaked irrigation fluid. This can induce hyponatremia in the patients. This fall in serum electrolytes is commonly followed by hypovolemia, with bradycardia and arterial hypotension. Hyponatremia is most pronounced 2–4 h later [15], but this extravasation may go undetected until the next day [16].

Generally, regional anesthesia should be performed during operations in which TUR syndrome might occur, because spinal anesthesia allows early detection of changes in mental status. However, the effectiveness of this approach is still controversial [17]. Symptoms of TUR syndrome may not be directly related to hyponatremia. Nevertheless, frequent blood sampling is required to accurately monitor serum sodium levels. Adding ethyl alcohol to the irrigation fluid may allow early detection of the absorption of irrigation fluid by permitting analysis of the alcohol content in the exsufflated air [18,19].

Hypotension during TURP can be caused by three factors: adopting a combination of epidural and spinal anesthesia; hyponatremia; and vagovagal reflex caused by filling of the bladder. It is difficult to distinguish between these phenomena. We carefully used the epidural anesthesia to avoid a sudden reduction in blood pressure. Continuous epidural anesthesia was used mostly with 5 ml/h of 0.2% ropivacaine. Vagovagal reflex may be judged as a cardiovascular abnormality.

Recently, the advent of bipolar electrodes for coagulation has allowed the use of electrolytic irrigation fluid [10]. Although this can decrease the risk of TUR syndrome, researchers have reported longer operative times owing to smaller and finer resection loops and a higher rate of postoperative urethral stricture [20,21]. Likewise, Holmium laser enucleation of the prostate (HoLEP) may become an alternative to standard TURP in the future. However, despite the reported decreases in bleeding, TUR syndrome remains a possibility with HoLEP [22].

## Conclusions

In this study, weight of resected mass > 45 g, volume of plasma substitute ≥ 500 ml, and continuous irrigation were associated with a significantly increased risk for TUR syndrome. Our results suggest that the use of a plasma substitute and continuous irrigation through a suprapubic cystostomy must be avoided during TURP procedures in the elderly to reduce the risk of TUR syndrome.

## Competing interests

The authors declare that they have no competing interests.

## Authors’ contributions

JN made substantial contributions to the conception, design and acquisition of data, and drafted the manuscript and tables. TS performed the statistical analysis and revised the manuscript critically for important intellectual content. AF participated in the design of the study and coordination and helped to draft the manuscript. TM made substantial contributions to the conception of the study and helped to draft the manuscript. All authors read and approved the final manuscript.

## Pre-publication history

The pre-publication history for this paper can be accessed here:

http://www.biomedcentral.com/1471-2253/14/30/prepub
